# How is AMSTAR applied by authors – a call for better reporting

**DOI:** 10.1186/s12874-018-0520-z

**Published:** 2018-06-18

**Authors:** Dawid Pieper, Nadja Koensgen, Jessica Breuing, Long Ge, Uta Wegewitz

**Affiliations:** 1 Institute for Research in Operative Medicine, Faculty of Health, School of Medicine, Witten/Herdecke University, Ostmerheimer Str. 200, 51109 Cologne, Germany; 2 The First Clinical Medical College of Lanzhou University, Lanzhou, 730000 China; 3 Evidence-Based Medicine Center, School of Basic Medical Sciences of Lanzhou University, Lanzhou, 730000 China; 4 Key Laboratory of Evidence-Based Medicine and Knowledge Translation of Gansu Province, Lanzhou, 730000 China; 5 Federal Institute for Occupational Safety and Health (BAuA), Nöldnerstr. 40-42, 10317 Berlin, Germany

**Keywords:** Systematic review, AMSTAR, Reporting, Methodological study, Quality assessment

## Abstract

**Background:**

The assessment of multiple systematic reviews (AMSTAR) tool is widely used for investigating the methodological quality of systematic reviews (SR). Originally, AMSTAR was developed for SRs of randomized controlled trials (RCTs). Its applicability to SRs of other study designs remains unclear. Our objectives were to: 1) analyze how AMSTAR is applied by authors and (2) analyze whether the authors pay attention to the original purpose of AMSTAR and for what it has been validated.

**Methods:**

We searched MEDLINE (via PubMed) from inception through October 2016 to identify studies that applied AMSTAR. Full-text studies were sought for all retrieved hits and screened by one reviewer. A second reviewer verified the excluded studies (liberal acceleration). Data were extracted into structured tables by one reviewer and were checked by a second reviewer. Discrepancies at any stage were resolved by consensus or by consulting a third person. We analyzed the data descriptively as frequencies or medians and interquartile ranges (IQRs). Associations were quantified using the risk ratio (RR), with 95% confidence intervals.

**Results:**

We identified 247 studies. They included a median of 17 reviews (interquartile range (IQR): 8 to 47) per study. AMSTAR was modified in 23% (57/247) of studies. In most studies, an AMSTAR score was calculated (200/247; 81%). Methods for calculating an AMSTAR score varied, with summing up all yes answers (yes = 1) being the most frequent option (102/200; 51%). More than one third of the authors failed to report how the AMSTAR score was obtained (71/200; 36%). In a subgroup analysis, we compared overviews of reviews (*n* = 154) with the methodological publications (*n* = 93). The overviews of reviews were much less likely to mention both limitations with respect to study designs (if other studies other than RCTs were included in the reviews) (RR 0.27, 95% CI 0.09 to 0.75) and overall score (RR 0.08, 95% CI 0.02 to 0.35).

**Conclusions:**

Authors, peer reviewers, and editors should pay more attention to the correct use and reporting of assessment tools in evidence synthesis. Authors of overviews of reviews should ensure to have a methodological expert in their review team.

## Background

Systematic reviews (SRs) synthesize the results of primary studies for a range of healthcare enquiries dealing with interventions, diagnosis, prognosis, etiology, and many other aspects [1]. Methodologically sound SRs are expected to provide the highest level of evidence for medical decision-making. During recent years, the assessment of multiple systematic reviews (AMSTAR) tool has become the most widely used tool for investigating the methodological quality of SRs [2, 3]. It was developed based on the Overview Quality Assessment Questionnaire [4, 5] and the checklist by Sacks [6] and consists of 11 items, each of which is categorized into a standardized set of four possible responses: “yes,” “no,” “can’t answer,” or “not applicable.” The items relate to a priori design, study selection and data extraction, the literature search, gray literature, the list of included and excluded studies, study characteristics, critical appraisal, formulation of conclusions, the combination of study results, publication bias, and conflicts of interest. It is important to note that a revised AMSTAR tool, called AMSTAR 2, was published in 2017 [7]. Due to its novelty, it has yet to be regularly applied in practice.

According to the developers of AMSTAR, the tool can be applied to a wide variety of SRs, although they recognize that its original development only took into account the SRs of randomized controlled trials (RCTs) for evaluating treatment interventions [8, 9]. This is not only true for the development of AMSTAR, but also for the content of the subsequent validation studies [8, 10]. The measurement properties of AMSTAR were recently described in an SR and found to be satisfactory in terms of inter-rater reliability, validity, and applicability [11]. Some of the included studies only considered observational studies. Interestingly, none of them mentioned that this was not the primary purpose of AMSTAR. This led to the hypothesis that many authors might not be aware of this fact. Furthermore, there is a debate on whether an overall score should be calculated as this was not mentioned as a possibility in the first AMSTAR development paper [9].

For all measurement instruments, it is important to focus attention to the tool’s measurement properties. When there are several potential measurement instruments available, the measurement properties might determine the choice of instrument. However, it should be noted that measurement properties might be context sensitive [12]. In the context of AMSTAR, this means that it might be questioned whether AMSTAR can be applied outside of its original scope (i.e., for SRs of RCTs for evaluating treatment interventions) without any limitations. Authors should at least be aware of this being a potential limitation.

Our objectives were to: 1) analyze how AMSTAR is applied by authors and (2) analyze whether the authors pay attention to the original purpose of AMSTAR and for what it has been validated.

## Methods

This study adheres to the preferred reporting items for systematic review and meta-analysis (PRISMA) [13]. As PRISMA aims to guide the reporting for SRs evaluating therapeutic efficacy, we deviated from the original checklist by omitting items due to the methodological focus of our study.

There was no a priori protocol for this study.

We performed a systematic literature search to identify relevant publications that have applied AMSTAR to at least one included study. Although we did not limit our search to a specific type of publication, we expected mainly two groups of publications to show up based on our experience in this field. The first group consisted of overviews of reviews, where AMSTAR is often used to assess the methodological quality on the included reviews [14]. The second group consisted of studies dealing with the methodological quality of SRs [15, 16]. We searched MEDLINE (via PubMed) from inception through October 2016. We searched for the terms “assessment of multiple systematic reviews” or “AMSTAR” in the title or abstract. No language restrictions were applied.

Instead of a conventional screening for titles and abstracts, we decided to retrieve the full texts of all potential articles that resulted from the literature search as we expected most of the retrieved hits to be included. All full texts were screened by one review author (DP, NK, or LG); those deemed not relevant were verified by a second person (DP, NK, or JB) for exclusion (liberal acceleration, also known as the safety first approach [17]). Discrepancies were resolved by consensus or by consulting a third person (DP or UW).

The data were extracted into structured Microsoft Office Excel sheets by one author and were checked for accuracy and completeness by a second author. Data were extracted on the study authors, study type, study designs included in the SRs, cited references related to AMSTAR (e.g., source paper), procedure of AMSTAR assessment (e.g., independently by two reviewers), number of reviewers (in total), number of reviewers per review, and inter-rater reliability (IRR) (if reported). We also recorded whether authors used the original AMSTAR version [9] or whether they used the modified version including notes to each item [18]. This latter version includes scoring guidance in the form of notes on each item. Furthermore, we collected data on any modifications made to AMSTAR by the authors, their rationale for using AMSTAR, and whether they used an overall score, and, if so, how it was calculated. Finally, we extracted whether reviews were categorized based on their overall score (if so, how), whether an overall score was used as an inclusion criterion, and whether the authors discussed potential limitations when calculating an overall score or applying AMSTAR not solely to SRs of RCTs. We did not contact any study authors.

We analyzed the data descriptively as frequencies or medians and interquartile ranges (IQRs). As we hypothesized that there might be differences between overviews of reviews opposed to methods papers, we performed a subgroup analysis by study type (overview vs. all others). Associations between study type and other characteristics were quantified using the risk ratio (RR), with 95% confidence intervals. The RR was calculated as it is generally more interpretable than the odds ratio [19]. All descriptive analyses were conducted with Microsoft Office Excel 2010 (Microsoft Corporation, Redmond, WA, USA). Risk ratios were calculated with MEDCALC (www.medcalc.org/calc/relative_risk.php).

## Results

In total, 247 studies met our inclusion criteria. They included a median of 17 reviews (interquartile range (IQR): 8 to 47) per study. When separated into subgroups, overviews of reviews included a median of 12 reviews (IQR): 7 to 27), and the methodological reviews included a median of 31 reviews (IQR 12 to 109). The characteristics of the included studies can be found in Table 1. Most articles were overviews of reviews (154/247; 62%) dealing with different questions (e.g., interventions, risk factors), including 12 Cochrane overviews. More than one in three (84/247; 34%) dealt with methodological aspects, such as the methodological quality of the SRs in a given field.

**Table 1:** Characteristics of the included studies

Study type (*n* = 247)		
Overview	154	62%
Methodological	84	34%
Discordance	8	3%
Psychometric	1	0%
Study designs included in SRs (*n* = 247)		
Only RCTs	39	16%
Not only RCTs	142	57%
Not reported	66	27%
AMSTAR assessment (*n* = 247)		
Independently	163	66%
Verification	9	4%
Duplicate on sample	2	1%
Single	1	0%
Not reported	72	29%
Number of reviewers in total (*n* = 247)		
2	86	35%
> 2	12	5%
Not reported	149	60%
Number of reviewers per included review (*n* = 247)		
1	3	1%
2	180	73%
5	1	0%
Not reported	63	26%
IRR reported (*n* = 247)		
Yes	58	23%
No	189	77%
AMSTAR version (*n* = 247)		
Original	25	10%
Modified [18]	9	4%
Not reported	213	86%
AMSTAR modifications (*n* = 247)		
Yes	57	23%
No	190	77%
Rationale for AMSTAR (*n* = 246)		
Yes	23	9%
No	223	91%
AMSTAR score (*n* = 247)		
Yes	200	81%
No	47	19%
Calculating AMSTAR score (*n* = 200)		
Yes = 1	102	51%
Yes = 1 with modifications	8	4%
Percentage	17	9%
Other	2	1%
Not reported	71	36%
AMSTAR score as inclusion criterion (*n* = 200)		
Yes	22	11%
No	178	89%
Categorization (*n* = 247)		
Yes	132	53%
No	115	47%
limitations with respect to study designs mentioned (*n* = 142)		
Yes	14	10%
No	128	90%
limitations with respect to overall score mentioned (*n* = 200)		
Yes	18	9%
No	182	91%

Percentages may not add up to 100%, as they are rounded to the nearest percent

More than half of the included studies stated that they did not restrict their included reviews solely to RCTs (142/247; 57%). Only 16% (37/247) reported that they only included SRs of RCTs, while any information on included study designs in reviews was missing in 27% (66/247) of the studies.

### Number of reviewers applying AMSTAR

In most cases (163/247; 66%), AMSTAR was applied to included reviews by reviewers independently, while 29% (72/247) of the authors failed to report the procedure. Most studies did not report the total number of reviewers involved in the AMSTAR assessment (149/247; 60%). At least two reviewers were involved in 35% (86/247) and 5% (12/247) of studies, respectively. Inter-rater reliability of the AMSTAR assessment was reported in 58 out of 247 (23%) studies.

### AMSTAR version and modifications

In most studies, it was not clear which AMSTAR version was applied (213/247; 86%), while in 10% (25/247) and 4% (9/247%) of the studies, it was clear that the authors had used the original and the modified version, respectively. A rationale for choosing the AMSTAR was reported by 9% (23/247) of study authors. AMSTAR was further modified in 23% (57/247) of the studies.

### Overall score

In most studies, an AMSTAR score was calculated (200/247; 81%). The methods for calculating an AMSTAR score varied, with summing up all yes answers (yes = 1) being the most frequent option (102/200; 51%) followed by calculating a percentage where the denominator contained all applicable questions (17/200; 9%). More than one third of the authors failed to report how the AMSTAR score was obtained (71/200; 36%). In 11% (22/200) of the studies, the AMSTAR score was used as an inclusion criterion for the study.

### Categorization

Reviews were categorized with respect to their methodological quality in 53% (132/247) of the studies. This was more frequent if authors calculated an overall score (123/200; 62%) compared to cases where no overall score was obtained (9/47; 19%), corresponding to a RR of 3.21 (95% CI 1.77 to 5.84). The authors used several different methods to categorize reviews based on their AMSTAR score. The authors most frequently (41/132; 31%) used a categorization where categories of quality were determined as follows: low (score 0 to 4), medium (score 5 to 8), and high (score 9 to 11). The second most frequent (33/132; 25%) categorization method, which was employed by the Canadian Agency for Drugs and Technologies in Health (CADTH), determines categories of quality as follows: low (score 0 to 3), medium (score 4 to 7), and high (score 8 to 11) [20].

### Addressing limitations

Potential limitations, with respect to obtaining an overall score, were only mentioned in 18 of the 200 (9%) studies. Potential limitations with respect to the included study designs in the assessed reviews were mentioned in 10% of the included studies, where the authors either did not state which study designs were included or they reported that the reviews also included other study designs than RCTs (14/142). Among all the studies that calculated an overall score, authors mentioning potential limitations with respect to the AMSTAR score also more frequently reported potential limitations with respect to the included study designs in the assessed reviews (3/17), as compared to authors who did not mention any limitations with respect to the AMSTAR score (14/160), corresponding to a RR of 2.02 (95% CI 0.64 to 6.32). Studies that included only the SRs of RCTs were excluded from this analysis.

### Subgroup analyses

In a subgroup analysis, we compared overviews of reviews (*n* = 154) with methodological publications (*n* = 93) of our sample. The results are shown in Table 2. The overviews of reviews were more likely to report the study design used in their reviews (RR 1.54, 95% CI 1.26 to 1.87). No differences were observed for reporting the AMSTAR assessment, the number of reviewers, the number of reviewers per review, and whether an AMSTAR score was obtained. However, overviews of reviews more often failed to report how the AMSTAR score was calculated (RR 0.66, 95% CI 0.54 to 0.81). Furthermore, overviews of reviews were less likely to report on IRR (RR 0.34, 95% CI 0.21 to 0.55), AMSTAR version used (RR 0.64, 95% CI 0.35 to 1.18), modifications made to AMSTAR (RR 0.72, 95% CI 0.46 to 1.13), and the rationale for choosing AMSTAR (RR 0.46, 95% CI 0.21 to 1.02). The strongest associations were observed for mentioning limitations. Overviews of reviews were much less likely to mention both limitations with respect to study designs (if other studies than RCTs were included in reviews) (RR 0.27, 95% CI 0.09 to 0.75) and overall score (RR 0.08, 95% CI 0.02 to 0.35).

**Table 2:** subgroup analysis

	Risk overview (*n* = 154)	Risk all other (*n* = 93)	Risk ratio (95% CI)
Study designs included in SRs reported	130/154 (84%)	51/93 (55%)	1.54 (1.26 to 1.87)
AMSTAR assessment reported	103/154 (67%)	67/93 (72%)	0.93 (0.78 to 1.10)
Number of reviewers reported	61/154 (40%)	37/93 (40%)	1.00 (0.73 to 1.37)
Number of reviewers per review reported	114/154 (74%)	70/93 (75%)	0.98 (0.85 to 1.14)
IRR reported	21/154 (14%)	37/93 (40%)	0.34 (0.21 to 0.55)
AMSTAR version reported	18/154 (12%)	16/93 (17%)	0.64 (0.35 to 1.18)
AMSTAR modifications	31/154 (20%)	26/93 (28%)	0.72 (0.46 to 1.13)
Rationale for AMSTAR reported	10/154 (6%)	13/93 (14%)	0.46 (0.21 to 1.02)
AMSTAR score obtained	121/154 (79%)	79/93 (85%)	0.93 (0.82 to 1.04)
Calculation of AMSTAR score reported^a^	65/121 (54%)	64/79 (81%)	0.66 (0.54 to 0.81)
AMSTAR score as inclusion criterion^a^	21/121 (17%)	1/79 (1%)	13.71 (1.88 to 99.90)
Categorization	90/154 (58%)	42/93 (45%)	1.29 (1.00 to 1.68)
Limitations with respect to study designs mentioned (if other studies than RCTs were included in reviews)^b^	5/96 (5%)	9/46 (20%)	0.27 (0.09 to 0.75)
Limitations with respect to overall score mentioned^a^	2/121 (2%)	16/79 (20%)	0.08 (0.02 to 0.35)

^a^studies with no overall score removed from the denominator

^b^reviews including only RCTs were removed from the denominator

## Discussion

Overall, there is a vast difference of when AMSTAR is applied by authors to assess the methodological quality of reviews. Most striking is the finding that limitations of AMSTAR are often not mentioned by the authors. Although AMSTAR has been designed and validated for SRs of RCTs for evaluating treatment interventions, more than half of our included studies also applied AMSTAR to SRs of nonrandomized studies. We acknowledge that there might not have been a proper tool to account for this at the time that the authors were conducting their study, but we argue that authors should mention this as a potential limitation. Based on our analysis, we are not able to judge whether authors either simply did not mention this potential limitation or they were not aware of the original purpose of AMSTAR. It has already been discussed earlier whether authors do not investigate an article or tool, but simply rely on “more informative titles” (MITs) [11]. AMSTAR has been described as a “reliable and valid measurement tool to assess the methodological quality of SRs” [10] without mentioning that it has only been investigated for use with SRs or RCTs. Furthermore, we acknowledge that most AMSTAR items are on the elements of which a review is composed (search, extraction, combining studies, publication bias etc.). These elements can be regarded to be relevant for all type of SRs, and thus not depending on the included type of studies in the SR. We are not aware of any study to which we could directly compare our study results. However, there are at least some hints from the urologic literature that authors overemphasize the terms *valid* and *reliable* while not being fully clear about its meaning and potential implications [21]. We expect such problems to be present in all fields of health research.

Many authors made modifications to AMSTAR. Although modifications might be necessary under certain circumstances, they should be kept to a minimum and a clear rationale should be provided. For example, Arevalo-Rodriguez et al. kept all the original questions of AMSTAR, while providing a detailed operationalization of it to make AMSTAR applicable to reviews of diagnostic test accuracy studies [22]. However, the modifications that authors have made to AMSTAR could also be interpreted that they were not fully satisfied with this tool. The potential limitations of AMSTAR have been debated in the literature, and modifications have been suggested by frequent AMSTAR users [23–25].

Another point of concern is the use of an overall score. The overall score was not mentioned in AMSTAR’s developmental paper [9], but only in the following validation papers [8, 10]. This has been described as meaningful for decision makers [10]. However, the developers also acknowledged that meta-analyses have a better chance of obtaining higher scores as not all items are relevant in the case of narrative synthesis. There is a lack of guidance about how AMSTAR should be scored in these cases. This explains our finding of authors using several ways to obtain an overall score. We admit that there might be situations where an overall score could be calculated for practical reasons. One example is the definition of cut-off points as an inclusion criterion, as we have seen in our sample. A recent study found that this does not introduce bias into overviews or reviews based on the finding that AMSTAR scores were not correlated with results/conclusions of reviews under study [14]. Another example is studies to validate or compare assessment tools, where an overall score might be helpful to allow for the calculation of measurement properties or correlations [11]. However, our results indicate that authors might not be aware of the potential limitations when using an overall score. The use of overall scores when assessing the quality of a study have already been debated in the literature [26, 27]. Presently, it seems well accepted that overall scores should not be used. This is in line with recently developed assessment tools for several study designs [28–30]. Significantly, the developers of AMSTAR 2 explicitly state that the calculation of an overall score is not intended in the updated assessment tool [7]. Instead, they provide a list of critical items that might be helpful to consider when rating the overall confidence in the results of a SR (high, moderate, low, critically low).

The problems of an overall score are perpetuated by categorizing reviews based on their AMSTAR scores. Authors used several methods for categorization, including possibilities to account for the abovementioned problems. For example, Joyce et al. applied different categorization schemes for SRs with and without meta-analyses given the fact that SRs with meta-analyses can obtain higher overall scores [31]. This important point could be neglected by authors when categorizing reviews because they will likely use the assigned category instead of the overall score. This might give too much strength to meta-analyses, as compared to SRs with narrative synthesis. Imagine two SRs scoring equally on all AMSTAR items, but item 10 that is related to the assessment of publication bias will only be answered “yes” for the SR with meta-analysis but not for the other. Applying the categorization of CADTH, the SR with meta-analysis could be judged to be of high methodological quality, while the second review will only be judged to be of medium methodological quality. In particular, this might have severe implications for overviews of reviews where evidence synthesis relies on SRs while paying attention to their methodological quality and/or risk of bias.

We hypothesized that there might be differences between overviews of reviews and methodological reviews applying AMSTAR for the assessment of SRs. Our results are in congruence with our prior expectations. Research teams of methodological reviews are better at reporting methodological aspects with respect to AMSTAR. Most importantly, authors of methodological reviews seem to be much more aware of potential limitations when applying AMSTAR. An explanation for this finding might be that methodological experts are more involved in methodological reviews, while overviews of reviews are more often written by context experts with limited methodological knowledge. According to the *Cochrane Handbook for Systematic Reviews of Interventions,* “review teams must include expertise in the topic area being reviewed and include, or have access to, expertise in systematic review methodology (including statistical expertise)” [32]. Overall, we have observed many shortcomings in reporting important methodological aspects that we consider to be important to allow for a thorough interpretation of study results.

Our work has some limitations. First, we did not perform a comprehensive literature search. However, despite having searched only one database, we included an extensive number of studies. This supports earlier findings that AMSTAR is frequently used to assess SRs [2, 3, 15]. Our findings might not be generalizable to other databases, and we will have missed studies that did not mention AMSTAR in their title or abstract. Nevertheless, some of our sample characteristics are comparable to those of other studies in terms of quality/risk of bias assessment [1] and number of included studies per review/overview [2, 3, 16]. Second, we did not try to contact authors for clarification or explanation of their choices. We only focused on what was reported in our included studies. So, we are not able to finally reject or accept our hypothesis that authors are often not aware of relevant aspects regarding AMSTAR. Third, differences in applying AMSTAR might be related to the levels of expertise and experience of the users. We were not able to take this into account.

Recently, two new tools for the assessment of SRs have been published. In 2016, a tool to assess the risk of bias in SRs rather than in primary studies, called ROBIS (risk of bias in systematic reviews), was introduced [29]. More recently, a newly modified AMSTAR tool, called AMSTAR 2, was published [7]. In contrast to AMSTAR, AMSTAR 2 is designed for the assessment of SRs, including RCTs and nonrandomized studies. We think that our study results might well be extrapolated to other assessment instruments, i.e., instruments used outside of their original purpose/scope or unnecessarily modified to a high degree without providing a rationale and a lack of adequate reporting.

## Conclusions

Authors, peer reviewers, and editors should pay more attention to the correct use and reporting of assessment tools in evidence synthesis. Authors of overviews of reviews should have a methodological expert on their review team.

## Abbreviations

AMSTAR: Assessment of multiple systematic reviews
CADTH: Canadian Agency for Drugs and Technologies in Health
CI: Confidence interval
IRR: Inter-rater reliability
MIT: More informative title
PRISMA: Preferred reporting items for systematic review and meta-analysis
RCT: Randomized controlled trial
RR: Risk ratio
SR: Systematic Review
